# An extracellular cation coordination site influences ion conduction of *Os*HKT2;2

**DOI:** 10.1186/s12870-019-1909-5

**Published:** 2019-07-15

**Authors:** Janin Riedelsberger, Ariela Vergara-Jaque, Miguel Piñeros, Ingo Dreyer, Wendy González

**Affiliations:** 1 Centro de Bioinformática y Simulación Molecular, Facultad de Ingeniería, Universidad de Talca, Talca, Chile; 2 Robert W. Holley Center for Agriculture and Health, USDA-ARS, Cornell University, Ithaca, NY USA; 3 Millennium Nucleus of Ion Channels-Associated Diseases (MiNICAD), Santiago, Chile

**Keywords:** Ion channel, HKT, Sodium transport, Potassium transport, Ion coordination site, Plant, Structure-function

## Abstract

**Background:**

HKT channels mediate sodium uniport or sodium and potassium symport in plants. Monocotyledons express a higher number of HKT proteins than dicotyledons, and it is only within this clade of HKT channels that cation symport mechanisms are found. The prevailing ion composition in the extracellular medium affects the transport abilities of various HKT channels by changing their selectivity or ion transport rates. How this mutual effect is achieved at the molecular level is still unknown. Here, we built a homology model of the monocotyledonous *Os*HKT2;2, which shows sodium and potassium symport activity. We performed molecular dynamics simulations in the presence of sodium and potassium ions to investigate the mutual effect of cation species.

**Results:**

By analyzing ion-protein interactions, we identified a cation coordination site on the extracellular protein surface, which is formed by residues P71, D75, D501 and K504. Proline and the two aspartate residues coordinate cations, while K504 forms salt bridges with D75 and D501 and may be involved in the forwarding of cations towards the pore entrance. Functional validation via electrophysiological experiments confirmed the biological relevance of the predicted ion coordination site and identified K504 as a central key residue. Mutation of the cation coordinating residues affected the functionality of HKT only slightly. Additional in silico mutants and simulations of K504 supported experimental results.

**Conclusion:**

We identified an extracellular cation coordination site, which is involved in ion coordination and influences the conduction of *Os*HKT2;2. This finding proposes a new viewpoint in the discussion of how the mutual effect of variable ion species may be achieved in HKT channels.

**Electronic supplementary material:**

The online version of this article (10.1186/s12870-019-1909-5) contains supplementary material, which is available to authorized users.

## Background

Proper sodium (Na^+^)/potassium (K^+^) homeostasis is a crucial requirement for high yielding plant growth. Although Na^+^ ions can promote plant growth at low concentrations, they turn hazardous at increasing levels [1–3]. Due to similar physicochemical properties, Na^+^ ions can mimic functions of K^+^ ions, bridging periods of K^+^ shortage [4, 5]. However, at high concentrations, Na^+^ ions compete with K^+^ and cause K^+^ deficiency symptoms in plants, since Na^+^ mimics the K^+^ function only incompletely [6, 7].

Plants have developed sophisticated mechanisms to cope with salt stress. These systems aim to avoid high cytosolic Na^+^ concentrations in cells of plant shoots by compartmentalization and retrieval of Na^+^ from the xylem sap [8, 9]. HKT channels form one comprehensive family that is involved in Na^+^ usage and detoxification [10–13]. In monocots, like rice, two types of HKT channels exist: (i) class I-type channels, which are mainly Na^+^ selective, and (ii) channels of class II acting as Na^+^/K^+^ co-transporters [14, 15]. The transmembrane structure of HKT channels is composed of one subunit containing four transmembrane-pore-transmembrane (MPM) units, which group together and form the conduction pathway in the center of the protein. Among HKTs, there is a wide variety of transport kinetics, cation selectivity and rectification properties even within the two classes [16–21].

Several studies have demonstrated the interactive effect of different ion species for various HKT channels. In particular, the mutual effects of Na^+^ and K^+^ have been documented [18, 21, 22]. However, the mechanism by which ion species affect each other and modulate the ion transport process is so far not well understood. Initially, it was discussed that HKT channels form multi-ion pores [17, 21]. In this scenario, multiple ion binding sites exist in the pore allowing a coupled ion transport without additional conformational changes [23]. However, recently Böhm and colleagues experimentally demonstrated that in the Venus flytrap HKT1 a maximum of one ion at a time occupies the selectivity filter [24]. Therefore, the mechanism behind mutual ion species effects remains controversial.

In this study, we provide new functional-structural perspectives for the understanding of ion species-specific effects on ion conduction in HKT channels. We built a homology model of the class II-type *Oryza sativa* HKT channel *Os*HKT2;2 embedded in a lipid membrane, performed molecular dynamics simulations in the presence of Na^+^ and K^+^ ions and evaluated protein-ion interactions. Through systematic analysis, we identified a potential cation coordination site at the extracellular surface of the protein – a region that ions have to pass before entering the pore. The cation coordination site was characterized by computational studies and its functional importance for *Os*HKT2;2 was experimentally validated using electrophysiological methods. Additionally, analyses of in silico mutants further underpinned the experimental insights.

## Results

### Electrophysiological characterization of *Os*HKT2;2

*Os*HKT2;2 is one out of eight to nine HKT channels (depending on the cultivar) expressed in rice plants [14]. Members of the HKT family show remarkable functional diversity regarding ion selectivity, rectification properties and effect of external cation compositions [20]. To provide a solid basis for this study, we first characterized the functional properties of *Os*HKT2;2 expressed in *Xenopus leavis* oocytes using the Two-Electrode Voltage-Clamp (TEVC) technique.

**Fig. 1:**
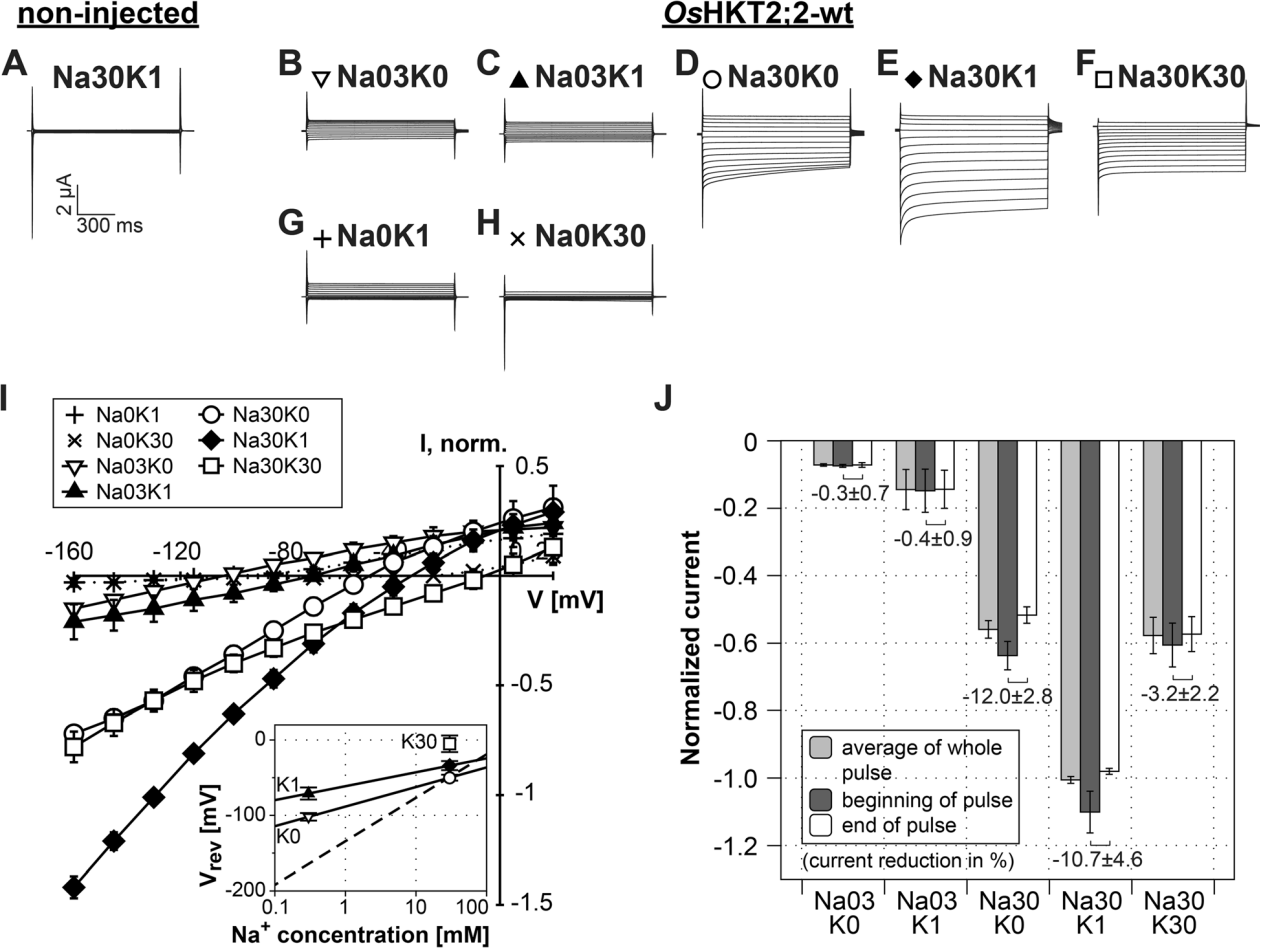
Electrophysiological characteristics of *Os*HKT2;2 in *Xenopus laevis* oocytes. **a** Representative currents elicited in control cells (not injected with cRNA). Only currents for Na30K1 solution are shown since highest currents are expected under these conditions. Control currents for all conditions are shown in Additional file 2: Figure S2. **b-h** Representative *Os*HKT2;2 mediated whole cell currents recorded two days after cRNA injection. Currents were recorded at indicated Na^+^ and K^+^ concentrations: Na0K1 - no NaCl and 1 mM KCl, Na0K30 - no NaCl and 30 mM KCl, Na03K0 - 0.3 mM NaCl without KCl, Na03K1 - 0.3 mM NaCl and 1 mM KCl, Na30K0 - 30 mM NaCl without KCl, Na30K1 - 30 mM NaCl and 1 mM KCl, Na30K30 - 30 mM NaCl and 30 mM KCl. The holding potential was set to the zero current level (approximately -45 mV with solution Na30K1) and 1 s voltage pulses were stepped from +20 to -160 mV in -15 mV decrements. A 1.5 s resting interval was allowed between successive voltage steps. The current (μA) and time (ms) scale for all traces shown in A through H is shown at the bottom of panel A. **i** Mean current-voltage (IV) curves. Standard deviation calculated from data obtained from five to eight measurements obtained on five different days and from four different oocyte batches. Currents were normalized to currents measured at -145 mV in Na30K1 solution. Inset: Reversal potentials (V_rev_) were determined based on the data plotted in I and plotted against the extracellular Na^+^ concentration. Symbols for V_rev_ and IV-curve data are identical and correspond to the same solutions in both plots. The external Na^+^ dependency of the V_rev_ values obtained with 1 mM KCl (filled triangle and diamond) or without KCl (empty triangle and circle) in extracellular solutions was fitted with a semi-logarithmic function (line K1 and K0). The dashed line represents the curve for an ideally Na^+^ permeable channel. **j** Currents elicited by a -145 mV voltage pulse, measured at the beginning (dark grey) or end (white) of the voltage pulse, and as average currents over the whole voltage pulse (light grey). Currents were normalized to currents measured in Na30K1 solution. Numbers in the plot indicate the degree of current reduction over time (from beginning to end of the voltage pulse). The magnitude of deactivation is stated in percentage. Means and standard deviation were obtained from data of five to eight measurements

Under voltage clamp conditions, cells expressing *Os*HKT2;2 conducted mainly Na^+^ inward currents in the absence of K^+^. Thereby, ion transport rates raised with increasing Na^+^ concentrations (Fig. 1b, d, i). At low extracellular Na^+^ concentrations (0.3 mM NaCl), ion currents of several hundred nanoamperes were detected, while at high Na^+^ concentrations (30 mM NaCl) currents increased to several microamperes consistent with Na^+^ being the ion carrying the current. A 100-fold increment of Na^+^ concentration (from 0.3 mM to 30 mM) resulted in an almost 5-fold increase of ion conduction.

The addition of low K^+^ concentrations (1 mM KCl) had a stimulating effect on the ion transport and led to a significant increase in ion conduction (Fig. 1d, e). One millimolar of KCl almost doubled the ion conduction at high and low Na^+^ concentrations. We are assuming a stimulating effect of K^+^ ions for two reasons: (1) In the absence of NaCl in the bath solution, no inward currents could be measured in the presence of 1 or 30 mM KCl (Fig. 1g, h). Currents at negative voltages were comparable to those recorded in control oocytes (compare Fig. 1g, h, i and Additional file 2: Figure S2). Therefore, potassium ions, at least in the absence of sodium ions, do not seem to pass the conduction pathway, or, they do pass with low velocity in a way the resulting currents cannot be clearly distinguished from background currents. (2) The stimulating effect was revoked under rising K^+^ concentration (30 mM NaCl/30 mM KCl; Fig. 1f, i, j). Interestingly, the ionic current measured with 30 mM NaCl/30 mM KCl was comparable to that observed without K^+^ ions in the extracellular solution indicating that K^+^ stimulates ion conduction only at low concentrations. Not only the increase of the Na^+^ concentration but also raising K^+^ concentrations provoked a shift of the reversal potential (V_rev_) towards less negative membrane voltages (inset of Fig. 1i, for the K^+^-dependency compare V_rev_ values in 0 mM (K0), 1 mM (K1) and 30 mM KCl (K30) at constant Na^+^), indicating that -besides Na^+^- also K^+^ ions may pass *Os*HKT2;2. The observation that despite this shift of V_rev_ the current amplitude only rises at low KCl concentrations in the media but not at high concentrations might be explained by different ion permeation velocities depending on the ion composition. While the presence of a few K^+^ ions could break electrostatic interactions between Na^+^ and the channel and thus might increase the ion mobility, elevated K^+^ concentrations could induce new hindering interactions and reduce ion mobility again.

Additionally, under high Na^+^ concentrations, inward currents partially deactivated at negative voltages. This effect was most pronounced in the absence of K^+^ and reduced with increasing K^+^ concentrations (Fig. 1d-f, j). At 30 mM NaCl (no KCl), the current amplitude was reduced by 12.0% (±2.8%) over one-second voltage pulses. In the presence of 1 mM KCl, the current reduction was 10.7% (±4.6%), and at 30 mM KCl current deactivation was almost abolished (3.2% ± 2.2%).

Overall, we identified and characterized three functional properties for wild-type *Os*HKT2;2 which we used later in the study to compare functional changes in mutated *Os*HKT2;2 channels (Table 1). The functional properties of wild-type *Os*HKT2;2 include: (1) ionic currents enhance with increasing Na^+^ concentration, (2) low K^+^ concentrations (1 mM KCl) stimulate Na^+^ ion transport, and (3) high K^+^ concentrations (30 mM KCl) lack the stimulating effect on Na^+^ ion transport.

**Table 1:** Electrophysiological characterisation of *Os*HKT2;2 and its mutants. The presence of three current characteristics are specified according to their degree of occurrence: very strong (+++), medium (++) or weak (+)

	Functionality	Currents enhance at rising Na^+^ concentration (Na03K0 vs. Na30K0)	Current increase with 1 mM K^+^	Lack of current enhancement at rising K^+^ concentration (Na30K0 vs. Na30K30)
wt		+++	+++	+++
P71A	like wt	+++	+++	+++
D75A	comparable to wt	++	+++	+++
D75N	comparable to wt	+++	+++	++
D501A	comparable to wt	++	+++	+++
D501N	comparable to wt	++	+++	++
K504R	altered kinetics	+	+++	+
K504Q	altered kinetics	+	+++	+
K504A	no conduction^a^			
K504E	no conduction			

^a^In some oocytes, very small currents could be detected 2–3 days after RNA injection. This mutant might function in a very inefficient manner so that currents accumulate to a measurable current only after long expression times. After two days of expression though, no currents comparable to wt or other mutants could be measured

### Identification of an extracellular cation coordination site

In order to identify amino acids that are approached by ions before entering the pore, we built a homology model of the *Os*HKT2;2 wild-type channel on the basis of the structurally and functionally related bacterial channels KtrB (PDB ID 4J7C) and TrkH (PDB ID 3PJZ). KtrB conducts Na^+^-dependent K^+^ transport for which reason its selectivity filter provides a good template for *Os*HKT2;2 that conducts both cations as well [25, 26]. Furthermore, it has been shown that KtrB alone (in absence of the regulatory KtrA unit) conducts K^+^ and Na^+^ ions, which further justifies the use of KtrB as template for homology modelling [27]. For structural comparison of template and model structure see Additional file 1: Figure S1. Since the N-terminus of KtrB is not included in the crystal structure, N-terminal amino acids of *Os*HKT2;2 were modeled on the basis of the TrkH (see Methods for details). Subsequently, we performed molecular dynamics (MD) simulation in the presence of 10 mM NaCl and 10 mM KCl for 100 ns. This allowed evaluating the frequency at which ions approached residues over time. To demonstrate reproducibility, the MD simulation was performed threefold and a total of 300 ns simulation time was reached, which formed the basis of the following results.

**Fig. 2:**
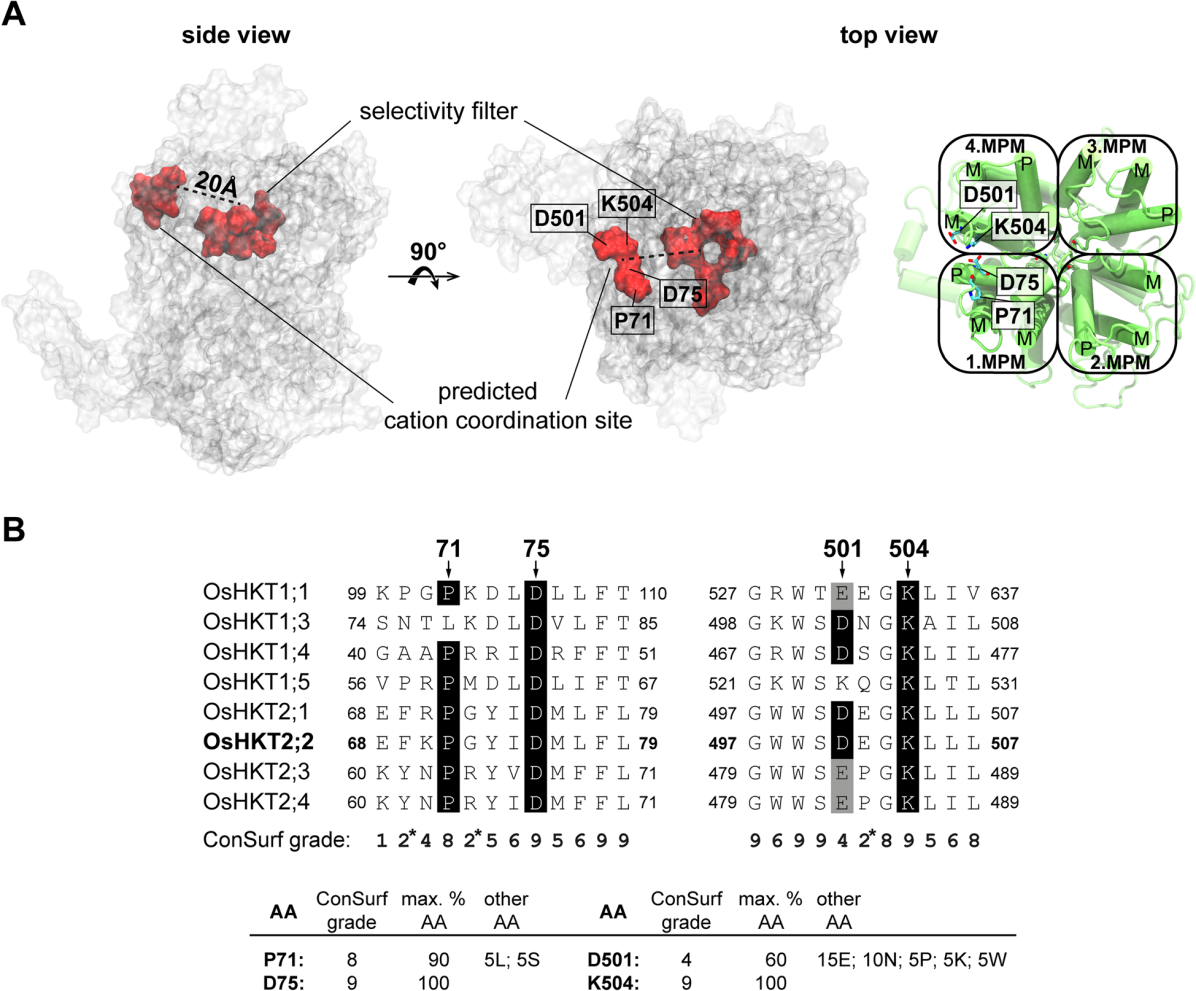
Localization and conservation of extracellular cation coordination site. **a** *Os*HKT2;2 is displayed as transparent surface representation with the selectivity filter and surrounding residues marked in the center of the channel as red surface representation. The cation coordination site is marked on the outer extracellular edge of the protein also as red surface representation, and the position of the four contributing residues is shown. The approximate distance between coordination site and pore is indicated in the top view representation. Detailed position of coordination site forming residues is illustrated on the right in the cartoon representation of *Os*HKT2;2. The four MPM units with their respective transmembrane (M) and pore domains (P) as well as P71, D75, D501 and K504 are marked. **b** An extract of the multiple sequence alignment of all eight HKT members present in *Oryza sativa*. Conservation of the residues P71, D75, D501 and K504 forming the coordination site is indicated. ConSurf grades were calculated on the basis of a multiple sequence alignment of 20 experimentally characterized HKT sequences, and are shown below the sequence alignment. The grades range from 1 (variable) to 9 (conserved) [29]. D75 and K504 are 100% conserved (ConSurf grade 9). P71 is in 90% of HKT sequences conserved (ConSurf grade 8), and contains in 5% of the sequences a lysine or serine. At position 501 a negative charge is somewhat conserved as aspartic in 60% of the sequences (ConSurf grade 4) or glutamic acid (in 15% of the sequences). In the remaining 25% of the sequences, this position holds an asparagine (10%), proline (5%), lysine (5%), or tryptophan (5%). Amino acid sequences used for this analysis are the following: *At*HKT1 (Q84TI7), *Ec*HKT1;1 (Q9LLM4), *Ec*HKT1;2 (Q9FVK0), *Hv*HKT2;1 (Q4VWZ3), *Mc*HKT1;1 (Q84UZ6), *Os*HKT1;1 (Q7XPF8), *Os*HKT1;3 (Q6H501), *Os*HKT1;4 (Q7XPF7), *Os*HKT1;5 (Q0JNB6), *Os*HKT2;1 (Q0D9S3), *Os*HKT2;2 (Q93XI5), *Os*HKT2;3 (Q8L481), *Os*HKT2;4 (Q8L4K5), *Pha*HKT2;1 (AB234305), *Put*HKT2;1 (C7B119), *Sl*HKT1;1 (CCJ09641), *Sl*HKT1;2 (CCJ09642), *Ta*HKT1;4 (A0N0D7), *Ta*HKT1;5 (A6XCE1), *Ta*HKT2;1 (Q41515)

Analyses of the contact frequency between ions and amino acids during MD simulations led to the identification of a potential extracellular cation coordination site (Table 2). This coordination site is located in the outer extracellular protein region approximately 20 Å away from the pore entrance (Fig. 2a). We identified three polar residues and one proline to be involved in the formation of a putative cation coordination site – P71, D75, D501, and K504. Within these, three are highly conserved among plant HKT channels (P71, D75, and K504), while at position D501 a negative charge is preserved in 75% of HKT channel sequences. Figure 2b illustrates the conservation of these four residues within the rice HKT family and additionally states their conservation within a multiple sequence alignment based on 20 experimentally characterized HKT channels.

**Table 2:** Contact frequencies of cations and selected amino acids. Absolute and relative frequencies of cations approaching (being within 4 Å) the four amino acids forming the potential extracellular cation coordination site (bold entries) and other amino acids in their proximity. Frequencies are based on 300 ns MD simulations and are displayed for sodium and potassium ions separately, respectively, as well as accumulated for both cations

	Sodium ions		Potassium ions		Sum of sodium and potassium ions	
Residue	Absolute frequency	Relative frequency (%)	Absolute frequency	Relative frequency (%)	Absolute frequency	Relative frequency (%)
F69	28	0.05	48	0.08	76	0.13
K70	306	0.50	273	0.45	579	0.95
**P71**	**1117**	**1.84**	**156**	**0.26**	**1273**	**2.10**
G72	690	1.14	134	0.22	824	1.36
Y73	12	0.02	0	0.00	12	0.02
I74	528	0.87	39	0.06	567	0.93
**D75**	**819**	**1.35**	**43**	**0.07**	**862**	**1.42**
M76	0	0.00	0	0.00	0	0.00
L77	0	0.00	0	0.00	0	0.00
W499	74	0.12	213	0.35	287	0.47
S500	97	0.16	322	0.53	419	0.69
**D501**	**1105**	**1.82**	**307**	**0.51**	**1412**	**2.33**
E502	2125	3.50	5042	8.31	7167	11.81
G503	0	0.00	12	0.02	12	0.02
**K504**	**900**	**1.48**	**125**	**0.21**	**1025**	**1.69**
L505	0	0.00	0	0.00	0	0.00
L506	0	0.00	1	0.00	1	0.00

### Structural attributes of the extracellular cation coordination site

**Fig. 3:**
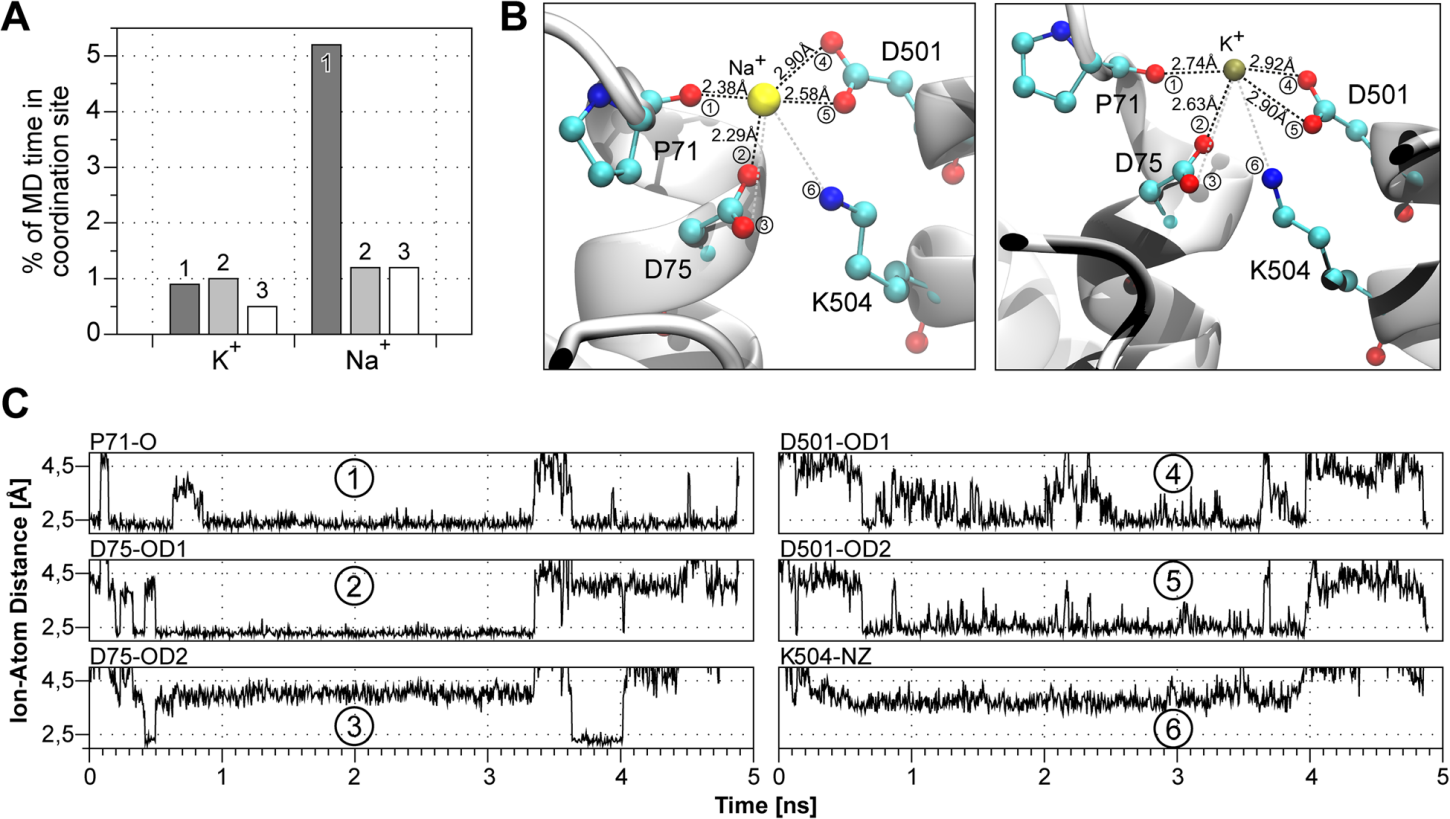
Oxygen atoms of P71, D75 and D501 coordinate cations. **a** Total time that K^+^ and Na^+^ stay in the coordination site during each of the three simulations expressed in percentage. **b** Coordination of cations happens via the backbone oxygen atom of P71, one side chain oxygen atom of D75 and both side chain oxygen atoms of D501 (red spheres). Average ion-atom distances for sodium and potassium are indicated in the figure. Sodium: 1) 2.38 ± 0.14 Å, 2) 2.29 ± 0.09 Å, 3) 4.02 ± 0.21 Å, 4) 2.90 ± 0.65 Å, 5) 2.58 ± 0.31 Å and 6) 3.74 ± 0.25 Å. Potassium: 1) 2.74 ± 0.19 Å, 2) 2.63 ± 0.30 Å, 3) 4.15 ± 0.40 Å, 4) 2.92 ± 0.47 Å, 5) 2.90 ± 0.38 Å and 6) 3.96 ± 0.28 Å. **c** Exemplary plot of ion-atom distances for sodium. Distances of one sodium ion and all six charged atoms facing the potential coordination site are represented for 5 ns of MD system 1 while Na^+^ is inside the pocket. The close proximity (< 2.5 Å) of Na^+^ to the backbone oxygen of P71, one side chain oxygen of D75 and both side chain oxygen atoms of D501 is illustrated, indicating their ability to coordinate sodium

Both sodium and potassium ions approached a defined area on the protein surface during MD simulations more often than residues in close proximity. K^+^ ions were found in this region between 0.5 to 1% of the simulation time, while Na^+^ stayed there about three times longer (1.2 to 5.2% of the time; Fig. 3a, Table 2). At this point, it is important to clarify that we were not searching for a region in the sense of an ion binding site that binds ions for extended time periods with potential allosteric impacts on protein function. Instead, we were searching for areas, which ions approach and pass on their way into the pore – in other words, coordination sites that attract cations and guide them to the pore entrance. This implicates that the total time of residence of an ion within the identified region is comparatively short as observed in our MD simulations. Nevertheless, cations approach the site frequently and are coordinated by the identified residues, in contrast to randomly approached residues where no ion coordination occurs. Throughout the study, we will refer to this region as extracellular cation coordination site.

Despite their short residence in the coordination site, the cations are coordinated by surrounding amino acids. A closer examination revealed four oxygen atoms coordinating the cations in this region: the backbone oxygen of P71 (Fig. 3b, c.1), one side chain oxygen atom of D75 (Fig. 3b, c.2) and the two side chain oxygen atoms of D501 (Fig. 3b, c.4 and c.5). The average distances between the mentioned oxygen atoms and Na^+^ were 2.38 Å, 2.29 Å, 2.90 Å and 2.58 Å, respectively, and, 2.74 Å, 2.63 Å, 2.92 Å and 2.90 Å for K^+^ (Fig. 3b). The coordination of one Na^+^ ion over time is exemplarily shown in Fig. 3c. Besides the proximity (< 2.5 Å) of the Na^+^ ion to the four mentioned oxygen atoms (plots c.1, c.2, c.4, c.5), the proximity to other side chain atoms facing towards the predicted coordination site is also shown. Namely, the proximity to the second side chain oxygen atom of D75 (plot c.3) and the positively charged nitrogen atom of K504 (plot c.6). The second side chain oxygen atom of D75 is oriented away from the coordination site most of the time and cannot contribute to the coordination of cations. As expected, the nitrogen of lysine 504 is also not involved in the coordination, since the positive charges of cations and the nitrogen atom repel each other.

**Fig. 4:**
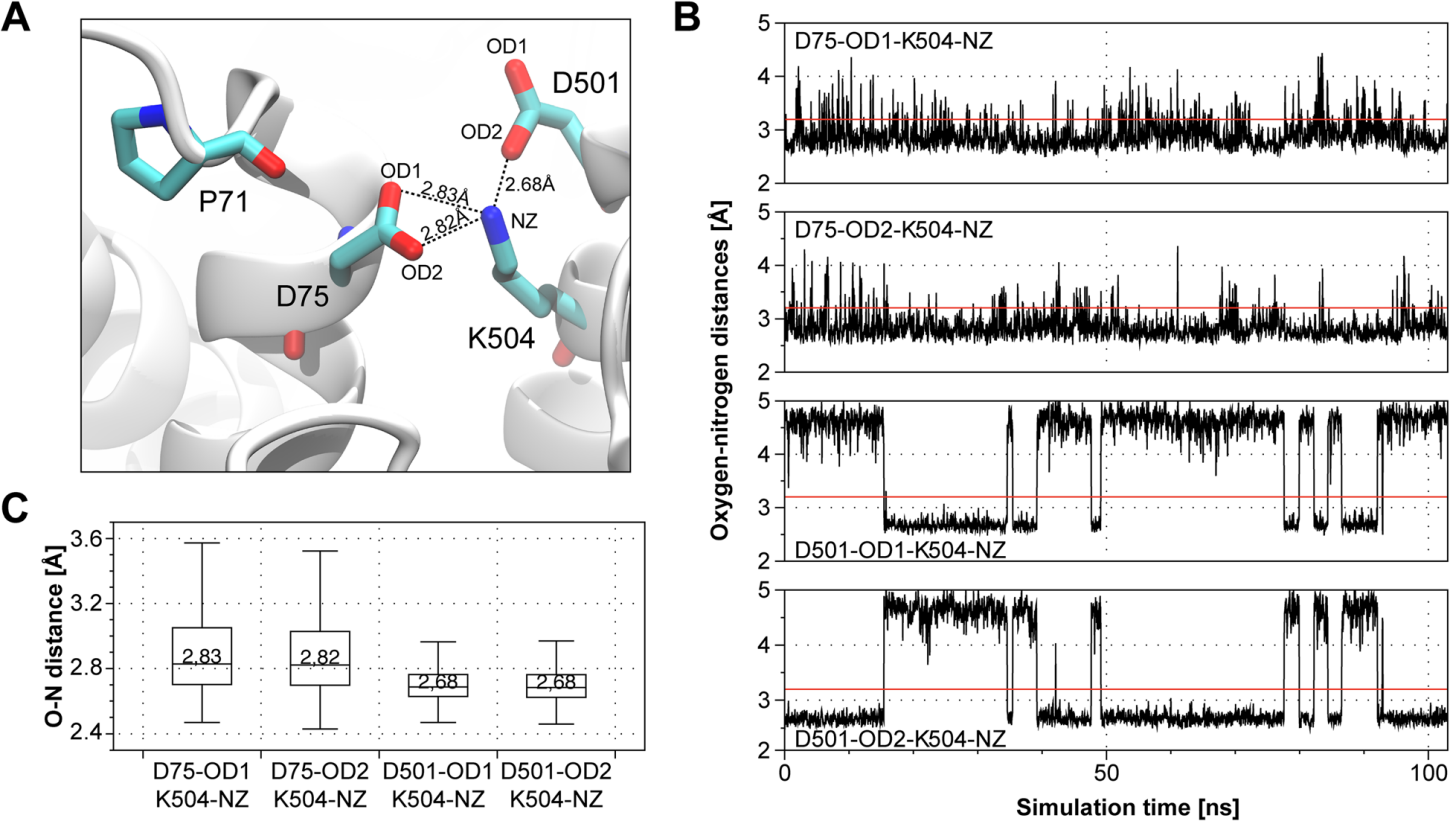
K504 can form salt bridges with D75 and D501. **a** Representation of potential salt bridges between K504 and D75 and D501. The median oxygen-nitrogen distance is stated. **b** Exemplary plot of oxygen-nitrogen distances throughout one complete simulation. The formation of salt bridges is considered possible when oxygen-nitrogen distances are below the threshold of 3.2 Å. The red line in each plot indicates this threshold. **c** Distance distribution of indicated oxygen-nitrogen distances over all three simulations. The median is stated in each box plot. Outliers are not drawn. For each of the two D501 oxygen atoms only those time spans where the respective oxygen atom was orientated towards K504’s nitrogen atom were used

Nevertheless, lysine 504 is located between the two aspartic acids D75 and D501, which suggests an interaction between these residues. Salt bridges may be formed between oppositely charged residues when the charged atoms are closer than 4 Å [28]. Average distances between the positively charged nitrogen atom of lysine 504 and the negatively charged oxygen atoms of both aspartates fulfill this requirement (Fig. 4a, b). Indeed, both oxygen atoms of D75 are in constant proximity to the nitrogen of lysine 504 (Fig. 4b, upper two traces) with an average distance of 2.83 Å and 2.82 Å, respectively (Fig. 4c). The two oxygen atoms of D501 alternate in their proximity to this nitrogen (Fig. 4b, lower two traces). If in proximity, the distance is 2.68 Å on average (Fig. 4c). Thus, lysine 504 may interact with both aspartates and acts as a structural component to obtain the geometry of the coordination site by keeping the aspartates in place rather than coordinating ions.

Overall, our computational studies suggest that the extracellular cation coordination site is constituted by a positively charged lysine (K504) that holds two negatively charged aspartates (D75 and D501) in place, which form a negative environment that attracts cations. The side chain oxygen atoms of both aspartates, in conjunction with the backbone oxygen of P71 coordinate Na^+^ and K^+^ ions.

### Experimental validation of the predicted cation coordination site

To study the relevance of the cation coordination site for the ion conduction of *Os*HKT2;2, we mutated P71, D75, D501, and K504 and experimentally characterized the changes in functionality in these mutants. Alanine mutations of all four residues were performed to assess the effect of side chain removal, and asparagine mutants of D75 and D501 allowed the evaluation of negative charge neutralization. Besides, K504 was mutated to arginine to examine the relevance of the side chain size, to glutamine to analyze the effect of charge neutralization, and to glutamic acid to evaluate the inversion from a positive to a negative charge. Overall, mutation of residue K504 had the most significant impact on *Os*HKT2;2’s functionality. Modifications of D75 and D501 affected the kinetic features slightly, whereas mutation of P71 did not cause changes in the transport mechanism as compared to the wild-type channel (Table 1).

### Mutations of P71, D75, and D501 show no or minor functional alterations

**Fig. 5:**
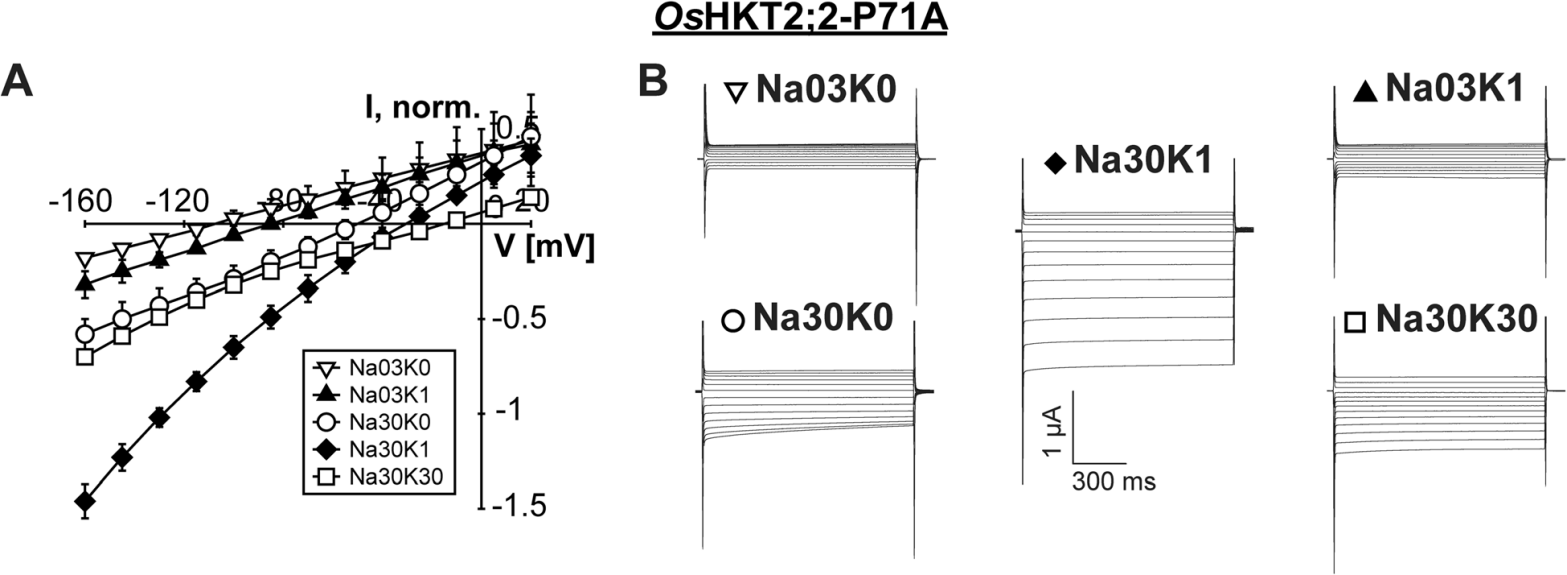
Mutation of P71 does not affect channel functionality. **a** Mean current-voltage (IV) curves and standard deviation calculated from data obtained from at least three different oocytes. **b** Representative currents measured at the indicated NaCl and KCl concentrations in *Xenopus laevis* oocytes two days after cRNA injection. A pulse at holding potential (zero current level) was followed by 1 s voltage pulses from +20 to -160 mV in -15 mV decrements and continued with a final pulse at holding potential for 1.5 s

To evaluate the effect of mutations of the cation coordinating residues we modified P71, D75 and D501 by the removal of side chains (alanine mutants) and charges (asparagine mutants). The mutant P71A behaved similarly to the wild-type channel regarding the three functional properties that have been established before on the basis of wild-type *Os*HKT2;2: Na^+^ conductivity (1), the mutual ion-species effect at low (2) and high (3) K^+^ concentrations (Table 1). P71A transported Na^+^ in the absence of K^+^, and the ion transport increased at high Na^+^ concentration. As observed in the wild-type channel, ion currents increased in the presence of 1 mM K^+^ but not 30 mM K^+^, when compared to currents recorded in the absence of K^+^ (Fig. 5).

**Fig. 6:**
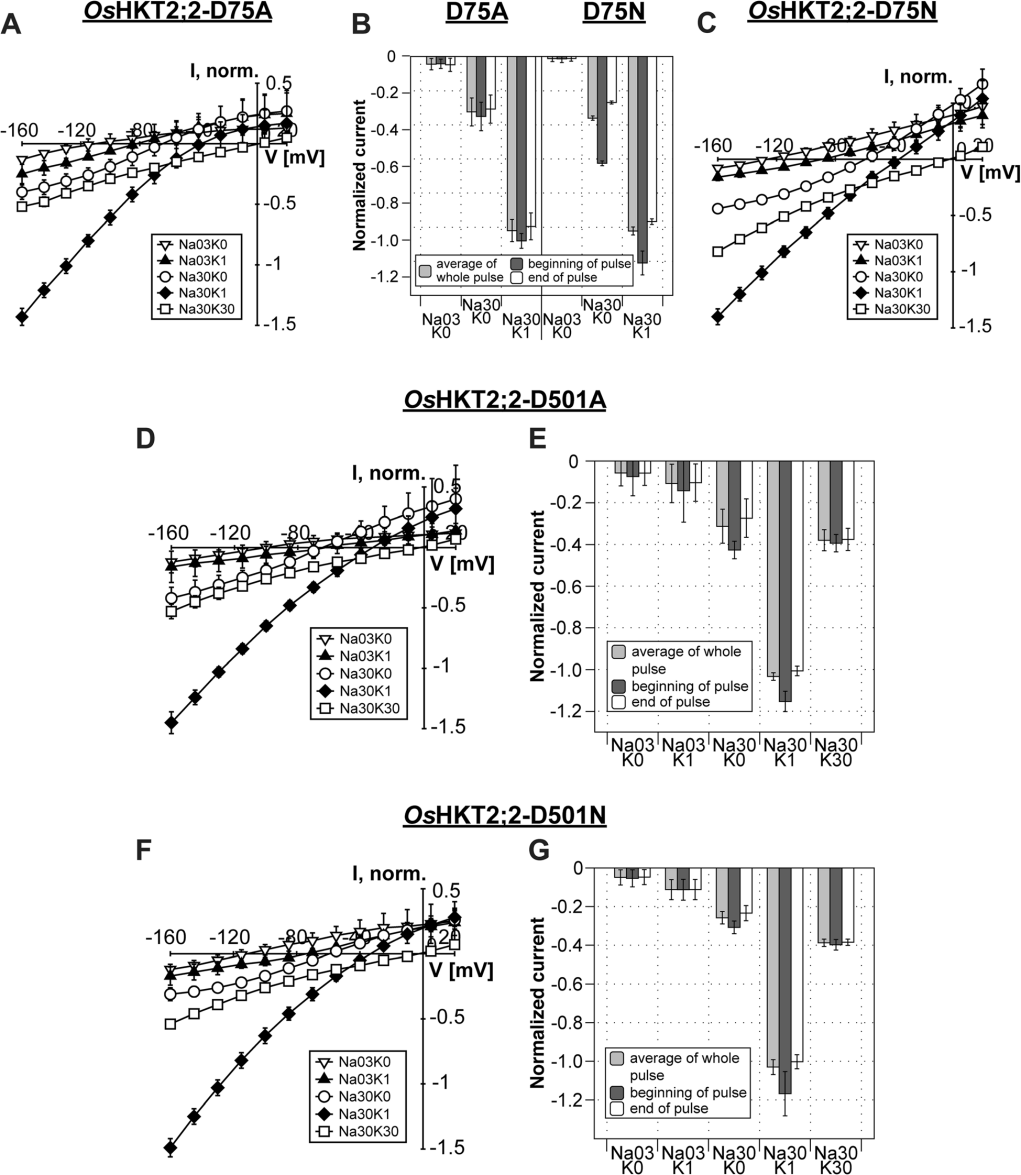
Mutations of D75 and D501 maintain channel functionality with only small changes in the kinetics. **a**, **c**, **d**, **f** Mean current-voltage (IV) curves and standard deviation calculated from data obtained from at least three different oocytes. **b**, **e**, **g** Currents measured at -145 mV at the beginning (dark grey) or end (white) of voltage pulses and of average currents over the whole voltage pulse (light grey) of D75A and D75N (**b**), D501A (**e**) and D501N (**g**). Currents were normalized to currents measured in Na30K1 solution. Means and standard deviations were obtained from data of three to five different oocytes

Mutations of residues D75 and D501 affected the ion conduction slightly. As observed in the wild-type channel, D75A/N and D501A/N transported Na^+^ in the absence of K^+^ in a concentration-dependent manner (0.3 mM NaCl and 30 mM NaCl). However, the increase of the current amplitude was slightly lower for D75A and D501A (4-fold), and only 3-fold in the D501N mutant, compared to a 5-fold increase for the wild-type channel (Fig. 6). The strong stimulating effect of low K^+^ concentrations on the ion conduction was observed in all mutants (30 mM NaCl/1 mM KCl). However, in contrast to wild-type *Os*HKT2;2, low magnitudes of current activation in the presence of high K^+^ concentrations (30 mM NaCl/30 mM KCl) were still observed in D75N and D501N mutants (Fig. 6c, f, g and Additional file 3: Figure S3) indicating that the mutual effect of K^+^ on Na^+^ currents had slightly shifted.

In summary, residues D75 and D501 were weakly sensitive towards modifications, while P71 was not being affected by the mutation. The latter was expected since proline coordinates cations via its backbone oxygen atom, which remains unaltered by mutations.

### K504 mutants showed substantial differences in their functionality compared to wild-type *Os*HKT2;2

**Fig. 7:**
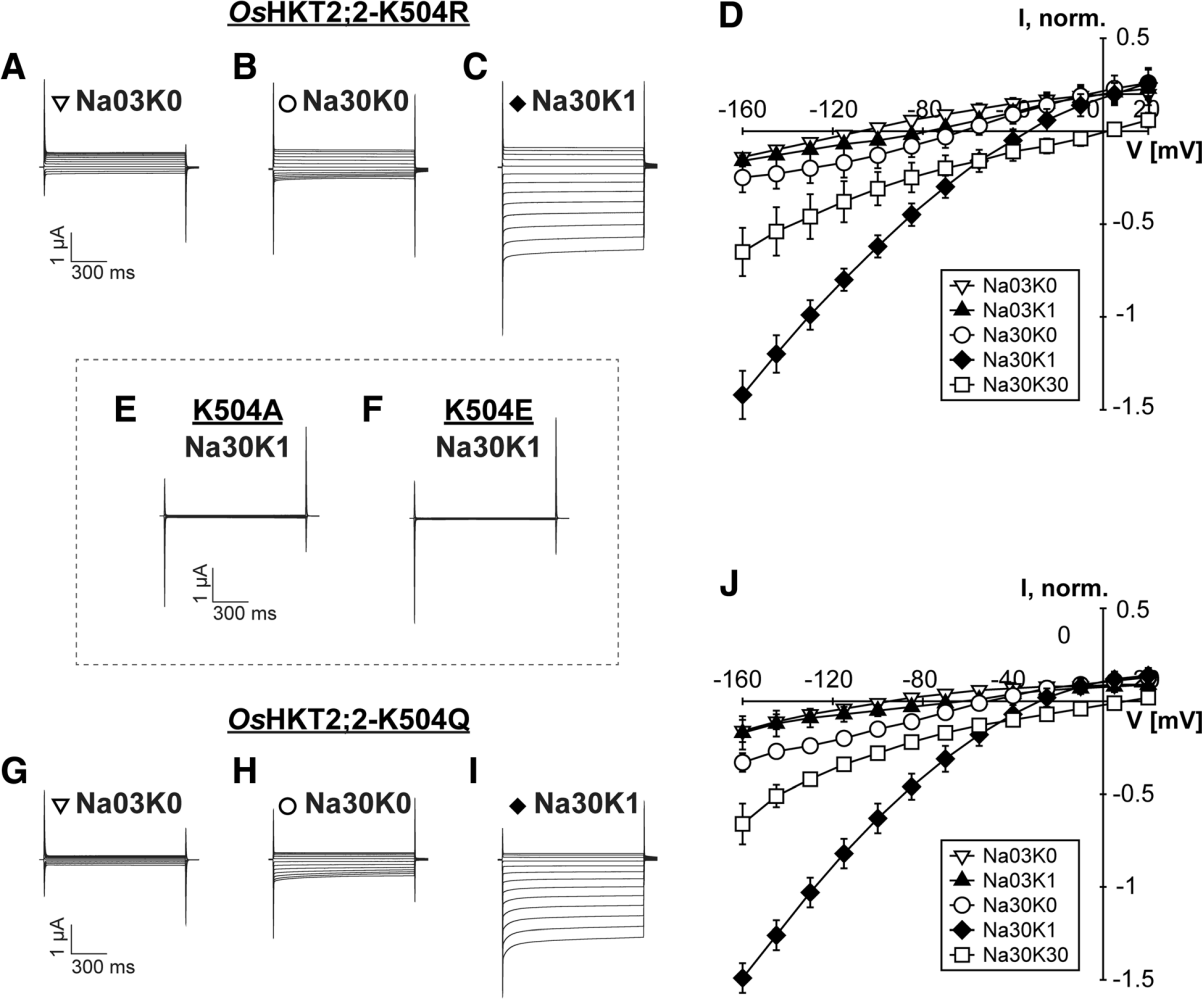
Mutations of K504 affect conduction behaviour of Na^+^ substantially. **a**-**c**, **e**-**i** Representative currents recorded in *Xenopus laevis* oocytes two days after cRNA injection at indicated Na^+^ and K^+^ concentrations: Na03K0 - 0.3 mM NaCl without KCl, Na30K0 - 30 mM NaCl without KCl, Na30K1 - 30 mM NaCl and 1 mM KCl. A pulse at holding potential (zero current level) was followed by 1 s voltage pulses from +20 to -160 mV in -15 mV decrements and continued with a final pulse at holding potential for 1.5 s. **d**, **j** Mean current-voltage (IV) curves and standard deviation calculated from data obtained from three to six measurements

Lysine 504 was identified as a structural component of the cation coordination site that seems to take a crucial position in the overall geometry. Indeed, K504 was very sensitive towards residue substitutions. Removal of the side chain (mutant K504A) and inversion of the charge (mutant K504E) rendered *Os*HKT2;2 in a manner that no ion conduction could be detected with the established measuring protocol. K504A and K504E injected oocytes showed no currents two days after cRNA injection (Fig. 7e, f). Each, fifteen injected oocytes have been analyzed on three different measuring days. However, oocytes injected with K504A cRNA sporadically conducted low currents in the nanoampere range, which differed significantly from those recorded in control oocytes. These observations were occasionally made three days after cRNA injection. We speculated that the K504A mutant may be functional but conducts ions with extremely low efficiency. In this case, the increased protein expression over time may lead to increased whole-cell currents through *Os*HKT2;2-K504A that are only detectable after long incubation times.

In contrast, lysine mutants K504R and K504Q were conducting currents although with substantial differences in their transport characteristics as compared to wild-type *Os*HKT2;2 and all other mutants. Na^+^ currents in the absence of K^+^ were low even at high Na^+^ concentrations (30 mM NaCl in comparison to 0.3 mM NaCl). In fact, the current amplitude rose not more than 2-fold with increasing Na^+^ concentration (compared to the 5-fold increase in wild-type channels) and remained in the nanoampere range. Therefore, the increase in the external Na^+^ concentration was not able to enhance the ion conduction to the same extent as in wild-type *Os*HKT2;2 (Fig. 7b, d, h, j). Regardless, low K^+^ concentrations did not only maintain its strong stimulating effect but also was this effect even more pronounced in the lysine mutants (30 mM NaCl and 30 mM NaCl/1 mM KCl, Fig. 7c, i). In the background of 30 mM NaCl, the addition of 1 mM KCl resulted in a more than 4- (K504Q) to 6-fold (K504R) increase of the current amplitude, while the wild-type was stimulated just about 2-fold. A further increase in the external K^+^ concentration was less inhibitory in the K504Q and K504R mutants than in the wild-type. While the wild-type showed almost identical current amplitudes at -160 mV measured in 30 mM Na^+^ and at 30 mM Na^+^ /30 mM K^+^, the amplitudes of the two mutants were about twice as large in 30 mM Na^+^ /30 mM K^+^ compared to those in 30 mM Na^+^ only (Fig. 7d, j and Additional file 4: Figure S4). This observation indicates that the Na^+^ transportability of *Os*HKT2;2 is affected in the K504R and K504Q mutants, while K^+^ ions are still able to promote and, in these mutants, also to rescue proper ion conduction.

### In silico mutations support experimental results

To gain further understanding of the nature of the experimental results, the four lysine mutants were studied in silico by performing 100 ns MD simulations. In each simulation, we examined the time that Na^+^ and K^+^ ions approached one of the four coordination site residues (< 4 Å) and the total number of approaches throughout the MD simulation (Table 3). The combined assessment of the duration of stay and number of approaches allowed the evaluation of the average time an ion spent in the cation coordination site. It is striking that Na^+^ ions approached the coordination site of the slow- or non-conducting lysine mutants K504A and K504E more frequently than in the conducting mutants K504Q and K504R (35 and 85 times versus 15 and 16 times; Table 3). The approaches observed in the mutants K504Q/R were comparable to the number of Na^+^ ion approaches in wild-type *Os*HKT2;2.

**Table 3:** Ion approaches of external coordination site in K504 in silico mutants compared to *Os*HKT2;2-wt. The proportion of time which cations stay within 4 Å of coordination site forming residues. Time is expressed as a percentage during 100 ns MD simulations. In addition, the number of approaches per ion species is indicated

(% of MD)	wt		K504R		K504Q		K504A		K504E	
Residue	Na^+^	K^+^	Na^+^	K^+^	Na^+^	K^+^	Na^+^	K^+^	Na^+^	K^+^
P71	1.9	0.3	0.5	1.4	6.0	4.4	1.9	0.3	3.2	1.0
D75	1.4	0.1	0.1	0.6	10.0	26.8	8.4	6.9	27.4	27.7
D501	1.8	0.5	2.5	3.2	12.9	13.8	9.6	7.3	16.0	11.6
K504X	1.5	0.2	0.2	0.4	8.0	21.2	0.02	–	24.0	38.7
No. of approaches	12	12	16	7	15	43	35	7	85	33

**Fig. 8:**
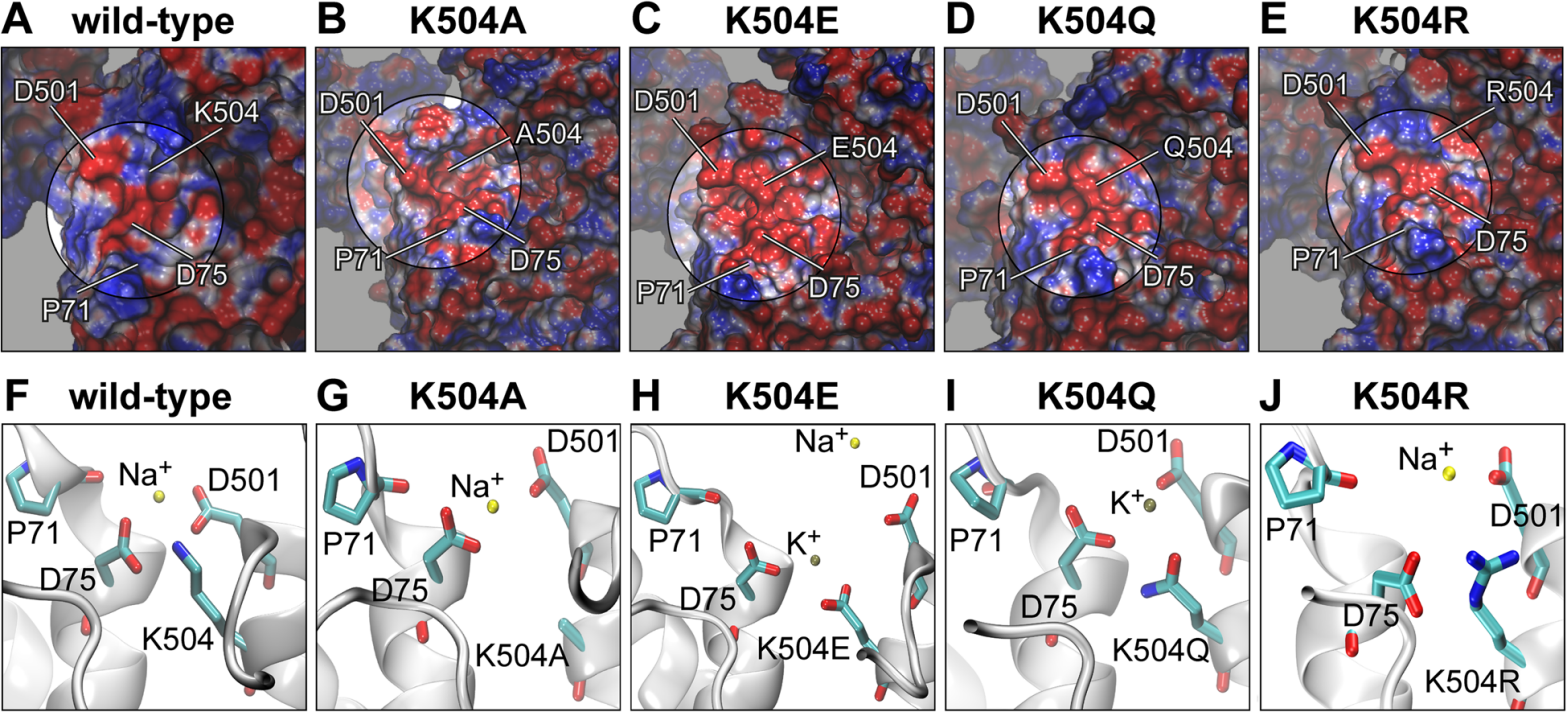
Coordination site constitution of in silico mutants K504A, K504E, K504Q and K504R. **a-e** Electrostatic potential mapped to the protein surface. The cation coordination site is highlighted and the position of P71, D75, D501 and K504 are indicated. **f-j** Representative illustration of the coordination site constitution of all four K504 mutants and the wild-type with ions approaching from the extracellular site or being inside the coordination site

In *Os*HKT2;2-K504R, where the positive charge was kept but the side chain slightly increased, Na^+^, as well as K^+^, approached the coordination site only shortly (see the short duration of stay and the low number of approaches in Table 3). Both cations were most of the time close to residue D501 (Na^+^: 2.5% and K^+^: 3.2% of simulation time), while in the wild-type the duration of stay was more balanced between the four coordination site residues. It is plausible that a more voluminous arginine residue at position 504 is preventing cations from entering into the coordination site due to the repulsion of positive charges. Although, the surface area around the coordination site is still negatively charged and able to attract cations, the positive charge at position 504 is bigger in the case of the mutant and further exposed to the extracellular space than in the wild-type channel, which may cause repulsion of cations (Fig. 8e, j compared to a, f).

In mutant K504Q, where the charge of the amino acid was neutralized, cations stayed at least five times longer in the coordination site (12.9 and 26.8% for Na^+^ and K^+^, respectively) than in mutant K504R, although both mutants showed comparable ion transport abilities in electrophysiological experiments. In contrast to mutant K504R, cations entered into the coordination site in mutant K504Q, which may account for the more extended stay in the pocket (compare Fig. 8i, j). Additionally, K^+^ ions approached the coordination site six times more frequently than in K504R, which may account at least in part for the longer duration of stay of K^+^ in the coordination site. Compared to the wild-type, cations stayed significantly longer close to the coordination site. The absence of the positive charge may allow cations to stay longer in the negatively charged coordination site before continuing to the pore entrance of the channel.

In the slow- or non-conducting mutants K504A and K504E, Na^+^ approached the coordination site with high frequency (35 and 85 times) and stayed up to 9.6% (K504A) and 27.4% (K594E) of the simulation time. K^+^ ions stayed shorter time than Na^+^ ions in the coordination site in mutant K504A (up to 7.3%) and longer in mutant K504E (up to 38.7%). The insertion of a negatively charged glutamate in mutant K504E generated a large negative area on the protein surface compared to *Os*HKT2;2-wt (Fig. 7c compared to a). A negative electrostatic potential highly attracts cations, and more than one cation approached the ion coordination site at a time, which may trap cations in the coordination site (Fig. 7g).

In summary, in silico data provide insights into potential reasons for functional alterations and the non-conduction of K504 mutants, respectively, and support experimental data.

## Discussion

HKT channels have been described as crucial players in salt tolerance. They transport Na^+^ as uniporter (class I) or in symport with K^+^ ions (class II). Class II-type HKT channels function as Na^+^ uniporter in the absence of K^+^. However, they become Na^+^/K^+^ symporter in the presence of K^+^. Analyses of the reversal potential suggest that K^+^ may be transported as well in the absence of Na^+^. However, it may be transported at such low velocity that resulting conduction cannot be distinguished from background currents in electrophysiological measurements. However, it has been shown in yeast complementation experiments that a K^+^ uptake deficient yeast strain could be complemented by expressing *Os*HKT2;2, and that K^+^ was depleted from the medium cultivating *Os*HKT2;2-expressing yeast [22]. Besides, K^+^ uptake by *Os*HKT2;2 was assessed in a plant expression system by analyzing rubidium uptake in tobacco protoplasts. Also here, a very low rubidium uptake could be detected in the absence of Na^+^ [21]. The mutual effect of ion-species on the ion conduction has been observed for several HKT channels. Dependent on the channel member the influence on ion transport varies [20]. Our electrophysiological studies confirmed the stimulating effect that K^+^ ions exert on Na^+^ transport in *Os*HKT2;2. Interestingly, this effect was only seen at low K^+^ concentrations and decreased as the extracellular K^+^ concentration increased. This phenomenon has been previously reported for *Os*HKT2;2 [21, 22]. From the physiological point of view, this is a consistent behavior. During K^+^ starvation (low K^+^ concentrations), ion uptake increases and with it the uptake of Na^+^ ions. Physico-chemically similar Na^+^ ions may complement functions of K^+^ up to a certain degree and may enable the survival of the plant while K^+^ availability is low. With increasing concentration of K^+^, the ion conduction is reduced and with it the uptake of Na^+^ ions. In the presence of sufficient K^+^ ions, high Na^+^ influx is not desired since Na^+^ ions compete with K^+^ and may cause symptoms of K^+^ starvation. However, while the physiological response of HKT is well investigated, the ion-specific effect at the molecular level is still unknown.

To get deeper insights into the molecular basis behind the mutual ion-species effect, we modeled the structure of *Os*HKT2;2 and analyzed cation-protein contacts. Our computational studies show that four residues P71, D75, D501 and K504 form an extracellular cation coordination site. Thereby, P71, D75 and D501 coordinate cations and the positively charged lysine (K504) holds the two negatively charged aspartates, D75 and D501, in place. Thus, a negative environment that attracts cations is formed.

We confirmed the relevance of the coordination site for the ion conduction of *Os*HKT2;2 in functional electrophysiological studies. Out of the three residues involved in ion coordination, residue D501 is slightly more sensitive towards modifications (see Table 1 for summary). D75 and P71 are located nearby on one extracellular end of a transmembrane helix and contribute to the cation coordination with each one oxygen atom. D501 is placed on the opposite side on the extracellular end of another helix and coordinates with two oxygen atoms (Fig. 2a, right). Therefore, mutating D501 removes all coordinating atoms from this side of the coordination site, while mutating D75 is only eliminating one of two coordinating atoms from the opposite side, which may account for the slightly stronger effect seen in D501 mutants. In either case, these mutations maintain the overall functionality of *Os*HKT2;2 and affect its kinetics only slightly. Considering this coordination site as one of many regions that ions pass before entering the pore, it is plausible that mutations affect the channel kinetics instead of rendering the channel non-functional. The identified coordination site would be involved in the attraction of cations and their forwarding towards the pore entrance, rather than in the tight and constant binding of ions. Therefore, modifying the coordinating oxygen atoms would first affect transport dynamics.

Lysine 504, on the other hand, is a crucial residue in the coordination site. Inverting the charge from positive to negative by a glutamate substitution results in a non-conductive mutant. With a third negative charge in the coordination site, the two aspartates repulse and drift apart, which leads to an expansion of the coordination site. Additionally, the three negative charges form a large negative region on the protein surface that attracts cations and traps them in the coordination site. If the identified coordination site is understood as one stopover for ions on their way into the pore, the trapped ions may explain the inhibited ion conduction, since they might block the path for ions leading into the pore.

Overall, the positively charged lysine 504 may be the necessary impulse that cations need to be forwarded towards the pore. The positive charge and moreover its correct size and position are crucial for channel functionality. The sensitivity towards changes of one of the parameters size or charge indicates how fine-tuned the constitution of the coordination site is. Removing the side chain by mutations to alanine renders *Os*HKT2;2 seemingly slow-conducting. Neutralizing the positive charge (K504Q) or increasing the size of the amino acid side chain with simultaneous maintenance of its charge (K504R), led to substantial changes in the conductance of *Os*HKT2;2. In the latter, the Na^+^ uniport is strongly reduced at high Na^+^ concentrations. A recent study of Xu and co-workers [30] showed that mutating G490 to arginine in HKT1;5 from *Triticum monoccocum* abolished its Na^+^ transport ability completely. G490 from *Tm*HKT1;5 is a highly conserved glycine among class I and class II HKT channels which corresponds to G503 in *Os*HKT2;2 and is the neighboring residue of K504 (analyzed in this study). The introduction of a voluminous positive charge in *Tm*HKT1;5-G490R is abolishing the Na^+^ transport, which supports our finding of a delicate constitution of the coordination site. Despite the reduced Na^+^ transport ability in K504 mutants, at low K^+^ concentrations, these mutants still mediate large ion currents indicating that either the coordination site still works correctly for K^+^ ions or that the positive effect of K^+^ ions has another origin. One possibility may be, e.g. the existence of another ion coordination site that is more relevant for the K^+^ forwarding than the one described here. In fact, during simulations, we could identify additional regions that are approached by ions on their way into the pore. These regions are currently under investigation.

The importance of K504 has been shown earlier for the wheat *Ta*HKT1 channel [31]. In this study, positively charged residues of the second transmembrane domain of the last MPM unit were mutated to investigate their contribution to ion conduction. Mutant K508Q, which corresponds to K504Q in our present study, showed reduced functionality in comparison to the wild-type channel in electrophysiological experiments and yeast complementation studies. Compared to wild-type *Ta*HKT1, *Ta*HKT1-K508Q complemented a K^+^ uptake deficient yeast strain although less effective than the wild-type. In electrophysiology experiments, the Na^+^ transport ability of *Ta*HKT1-K508Q was affected, a result that we also observed in *Os*HKT2;2-K504Q. The authors also proposed a potential salt bridge formation between K508 and D78 based on a homology model of *Ta*HKT1. *Ta*HKT1-D78 corresponds to D75 in *Os*HKT2;2, which we found to form salt bridges with K504, which is in agreement with the prediction made by Kato and colleagues. Those results and the ones presented in our study point to the functional relevance of K504 in combination with D75. Our study additionally provides a biological and structural context by identifying these residues as part of a cation coordination site.

## Conclusion

Overall, using computational methods, we identified an extracellular cation coordination site and validated its functional relevance experimentally. Although being about 20 Å far from the pore entrance, the identified coordination site is essential for proper ion conduction. Crucial ion coordination sites on the extracellular protein surface provide an example of how different ion species may affect the channel conduction behavior even before entering the pore. We are still far from understanding how K^+^ ions influence the ion transport of *Os*HKT2;2 on the molecular level. This study, however, provides new evidence, which opens the discussion for new explanations for the mutual effect of ion species.

## Methods

### Homology modelling and molecular dynamics simulation

The structural model of *Os*HKT2;2 (accession number: Q93XI5) was generated using the I-Tasser server [32]. Full-length structural models were built from multiple threading alignments. For threading, the bacterial channels KtrB (PDB ID 4J7C) and TrkH (PDB ID 3PJZ) were used. The mayor part of the channel was modeled on the basis of KtrB (residues 39–530), while the N-terminus of *Os*HKT2;2 (residues 1–38) was modeled on the basis of TrkH, since the crystal structure of KtrB does not contain the N-terminal region. I-TASSER calculates two values, which are recommended for evaluating model quality and correct folding. Model quality is estimated by the C-score, where a value higher than −1.5 indicates a generally correctly folded protein. The second value, the TM-value, gives a measure of structural similarity compared to the native structure. A TM-value higher than 0.5 indicates proteins of similar folds. C-score and TM-value of the best model generated by I-TASSER were − 0.4 and 0.66, respectively, which is above the threshold, and indicates that the generated model represents a correctly folded protein.

*Os*HKT2;2 contains a 29 amino acid long extracellular loop that is not present in bacterial channels. Due to a missing template, the loop spanning residues 473–501 were modeled ab initio by I-TASSER due to the missing template. To improve the model quality, we modeled that part with the FALC-Loop server, a program that is specialized in loop modeling, and which combines statistical and knowledge-based methods [33]. The improved full-length model was refined using i3Drefine [34] and minimized using CHARMM via its web portal CHARMMing [35, 36]. Throughout the whole process, model quality was monitored independently by quality assessment tools like Procheck [37], QMEAN [38] and ModFold [39, 40]. The refined model was embedded in a pre-equilibrated POPC bilayer solvated with pre-equilibrated TIP3P water molecules [41] in a periodic boundary condition box using VMD [42]. The system was neutralized by the addition of sixteen chloride ions. Each, six Na^+^ and K^+^ ions as well as twelve further chloride ions were added to mimic the concentration of 10 mM NaCl and 10 mM KCl. The entire system underwent several minimization steps and was equilibrated with successive restraint reduction until restraints were removed entirely to ensure step-by-step equilibration and avoid degeneration of the protein. Each reduction of restrictions was monitored by checking system temperature, pressure and energy, as well as, the RMSD value of the protein. The consequent reduction of restriction was not initiated unless the RMSD value was not stable. This procedure was continued until all restrictions were removed entirely and the RMSD converged to a stable value. Not until then, were the production runs of the MD simulations initiated. Three replicates of 100 ns molecular dynamics (MD) simulations were generated using NAMD [43, 44].

Coordination site residues were identified by counting the frames in which an ion was within 4 Å of a given residue during MD simulations. Ion coordination was examined by measuring distances between charged atoms of P71, D75, D501, K504 and the ion present in the coordination site. Salt bridges were evaluated using VMD’s Salt Bridge plugin. Electrostatic potentials were calculated using the APBS web server with the ionic strength set to simulate 10 mM NaCl and 10 mM KCl [45]. APBS input files were generated using the PDB2PQR Server version 2.1.1 [46, 47].

In silico mutations of *Os*HKT2;2 were generated via VMD’s Mutate plugin. Mutants were minimized and equilibrated followed by 15 ns of MD simulation in NAMD.

### Electrophysiology

Wild-type and mutant *Os*HKT2;2 constructs were cloned into the pNB1u vector for expression in *Xenopus laevis* oocytes using the USER-cloning technique [48]. Utilized primers were: GGCTTAAUatgacgagcatttaccaagaa (forward) and GGTTTAAUctaccatagcctccaatatt (reverse). Vector-specific and *Os*HKT2;2-specific sequences are given in uppercase and lowercase format, respectively. The position of uracil is underlined. Standard fusion PCR technique was used for site-directed mutagenesis. After DNA linearization with *NotI*, cRNA was synthesized using the mMessage mMachine in vitro transcription kit following the manufacturer’s guidelines.

For expression in oocytes, stage V and VI oocytes were harvested from *Xenopus laevis* and kept in ND96 solution (96 mM NaCl, 2 mM KCl, 1.8 mM CaCl_2_, 1 mM MgCl_2_, 2.5 mM Na Pyruvate, 5 mM Hepes – pH 7.5, 50 mg mL^− 1^ gentamycin and 0.4 g L^− 1^ BSA). Oocytes were defolliculated by collagenase treatment in ND96 without CaCl_2_, gentamycin and BSA and kept overnight at 18 °C in complete ND96 solution as described initially. All animal procedures, including husbandry, oocyte harvesting and post-treatment, were performed in accordance with Cornell University IACUC Protocol number 2017–0139. Animals were purchased from Xenopus Express Inc. (Brooksville, FL, USA). 50 nl of cRNA (500 ng/μl) were microinjected into oocytes using an oil-driven injection system (Nanoject II Auto-Nanoliter Injector, Drummond Scientific Company, US). Cells were then incubated for two days at 18 °C in complete ND96 solution. Whole-cell currents were recorded using conventional Two-Electrode Voltage-Clamp technique (GeneClamp 500 amplifier and Digidata 1320A-PClamp 10 data acquisition system, Axon Instruments). Recordings were carried out under constant perfusion of bath solutions containing 2 mM MgCl_2_, 1.8 mM CaCl_2_, 10 mM MES, pH 5.5 with Tris-Base with the addition of: 1 mM KCl and 165 mM Sorbitol (Na0K1); 30 mM KCl and 130 mM Sorbitol (Na0K30); 0.3 mM NaCl and 165 mM Sorbitol (Na03K0); 0.3 mM NaCl, 1 mM KCl and 165 mM Sorbitol (Na03K1); 30 mM NaCl and 130 mM Sorbitol (Na30K0); 30 mM NaCl, 1 mM KCl, and 130 mM Sorbitol (Na30K1); 30 mM NaCl, 30 mM KCl and 100 mM Sorbitol (Na30K30).

## Additional files

Additional file 1: **Figure S1.** Structural comparison of KtrB template and *Os*HKT2;2 model. Template and model structure were aligned using the structural alignment algorithm TM-align [49]. A TM-score of 0.90536 was calculated indicating that both structures have the same fold. (**A**) Overlay of KtrB (PDB ID 4J7C) (purple) and modeled *Os*HKT2;2 (green) in side and top view. Alpha helices are displayed as tubes. Residues forming the selectivity filter and cation coordination site are represented in licorice. Selectivity filter forming residues in (1) KtrB: G62, G177, G280 and G392, (2) *Os*HKT2;2: G88, G244, G368 and G469. Cation coordination site forming residues in *Os*HKT2;2: P71, D75, D501 and K504. Corresponding residues in KtrB: L45, D49, T403 and K406. (**B**) Closer top view on cation coordination site (left) and selectivity filter (right). Positions of respective residues in the structure are illustrated by schematic representations to the left and right of the zoom. (TIF 7748 kb)

Additional file 2: **Figure S2.** Currents elicited in control cells. (**A**) Representative currents recorded in *Xenopus laevis* oocytes at indicated Na^+^ and K^+^ concentrations: Na03K0 - 0.3 mM NaCl without KCl, Na03K1 - 0.3 mM NaCl and 1 mM KCl, Na30K0 - 30 mM NaCl without KCl, Na30K1 - 30 mM NaCl and 1 mM KCl, Na30K30 - 30 mM NaCl and 30 mM KCl. Control oocytes underwent the same handling and incubation procedure as injected oocytes and were measured on the same day as cRNA injected oocytes. A pulse at holding potential (zero current level) was followed by 1 s voltage pulses from +20 to -160 mV in -15 mV decrements and continued with a final pulse at holding potential for 1.5 s. (**B**) Representative current-voltage (IV) curves extracted form current traces shown in (A). For comparison the mean IV curve of *Os*HKT2;2-wt from Fig. 1g is presented (x). (TIF 574 kb)

Additional file 3: **Figure S3.** D75A, D75N, D501A and D501N mutants behave comparable to *Os*HKT2;2-wt. Representative currents recorded in *Xenopus laevis* oocytes two days after cRNA injection at indicated Na^+^ and K^+^ concentrations: Na03K0 - 0.3 mM NaCl without KCl, Na03K1 - 0.3 mM NaCl and 1 mM KCl, Na30K0 - 30 mM NaCl without KCl, Na30K1 - 30 mM NaCl and 1 mM KCl, Na30K30 - 30 mM NaCl and 30 mM KCl. A pulse at holding potential (zero current level) was followed by 1 s voltage pulses from +20 to -160 mV in -15 mV decrements and continued with a final pulse at holding potential for 1.5 s. (TIF 1307 kb)

Additional file 4: **Figure S4.** Mutants K504R and K504Q show altered kinetic characteristics in comparison to *Os*HKT2;2-wt. Representative currents recorded in *Xenopus laevis* oocytes two days after cRNA injection at indicated Na^+^ and K^+^ concentrations: Na03K0 - 0.3 mM NaCl without KCl, Na03K1 - 0.3 mM NaCl and 1 mM KCl, Na30K0 - 30 mM NaCl without KCl, Na30K1 - 30 mM NaCl and 1 mM KCl, Na30K30 - 30 mM NaCl and 30 mM KCl. A pulse at holding potential (zero current level) was followed by 1 s voltage pulses from +20 to -160 mV in -15 mV decrements and continued with a final pulse at holding potential for 1.5 s. (TIF 638 kb)

## Data Availability

All data generated or analyzed during this study are included in this published article and its supplementary information files.
