# Supplementary figures and images for: An extracellular cation coordination site influences ion conduction of OsHKT2;2
Source: BMC Plant Biol. 2019 Jul 15;19:316. doi: 10.1186/s12870-019-1909-5 (PMC6632200; doi:10.1186/s12870-019-1909-5)

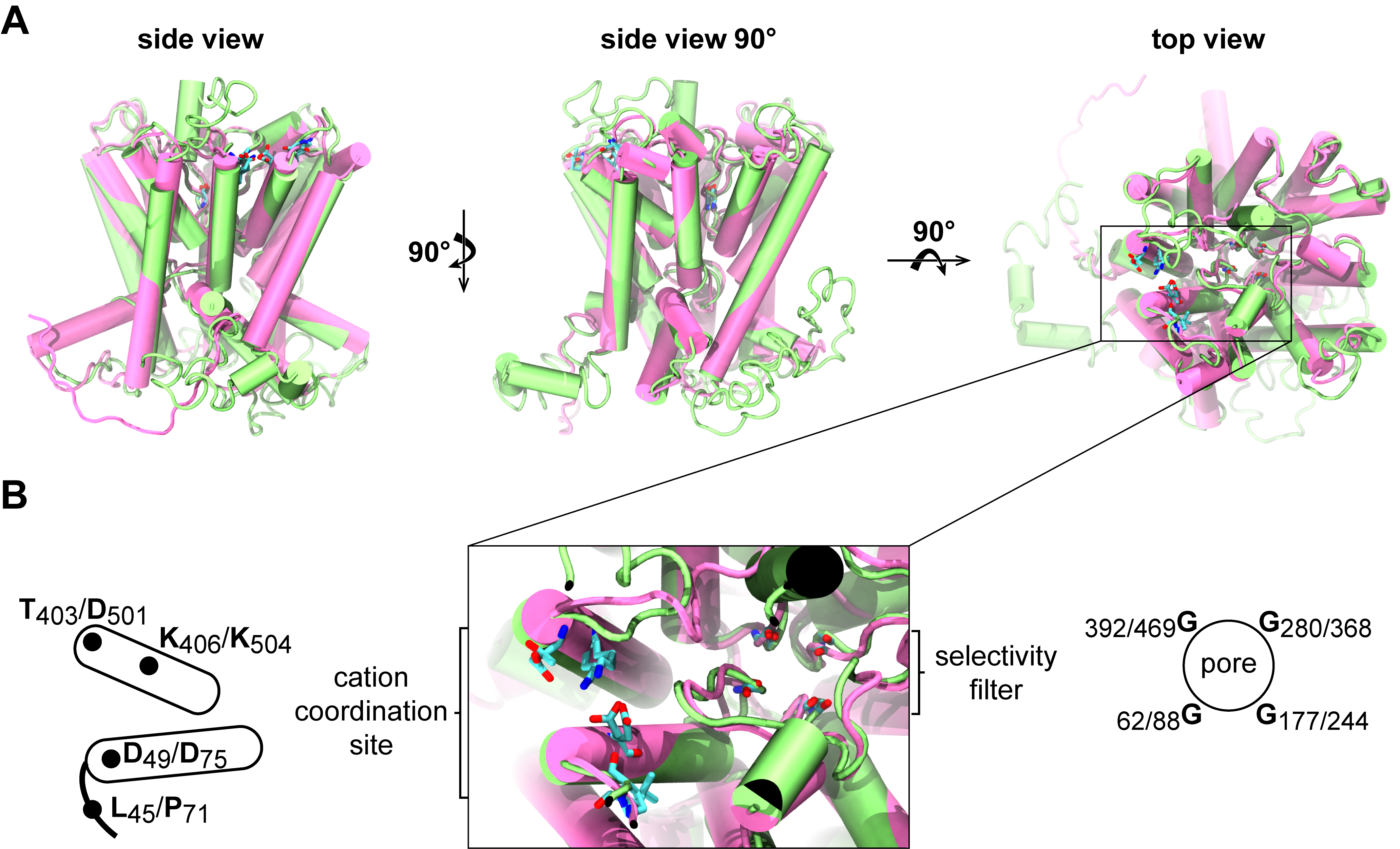

Supplement: Supplementary file 1 — Figure S1. Structural comparison of KtrB template and OsHKT2;2 model. Template and model structure were aligned using the structural alignment algorithm TM-align [49]. A TM-score of 0.90536 was calculated indicating that both structures have the same fold. (A) Overlay of KtrB (PDB ID 4J7C) (purple) and modeled OsHKT2;2 (green) in side and top view. Alpha helices are displayed as tubes. Residues forming the selectivity filter and cation coordination site are represented in licorice. Selectivity filter forming residues in (1) KtrB: G62, G177, G280 and G392, (2) OsHKT2;2: G88, G244, G368 and G469. Cation coordination site forming residues in OsHKT2;2: P71, D75, D501 and K504. Corresponding residues in KtrB: L45, D49, T403 and K406. (B) Closer top view on cation coordination site (left) and selectivity filter (right). Positions of respective residues in the structure are illustrated by schematic representations to the left and right of the zoom. (TIF 7748 kb) [file 12870_2019_1909_MOESM1_ESM.tif]

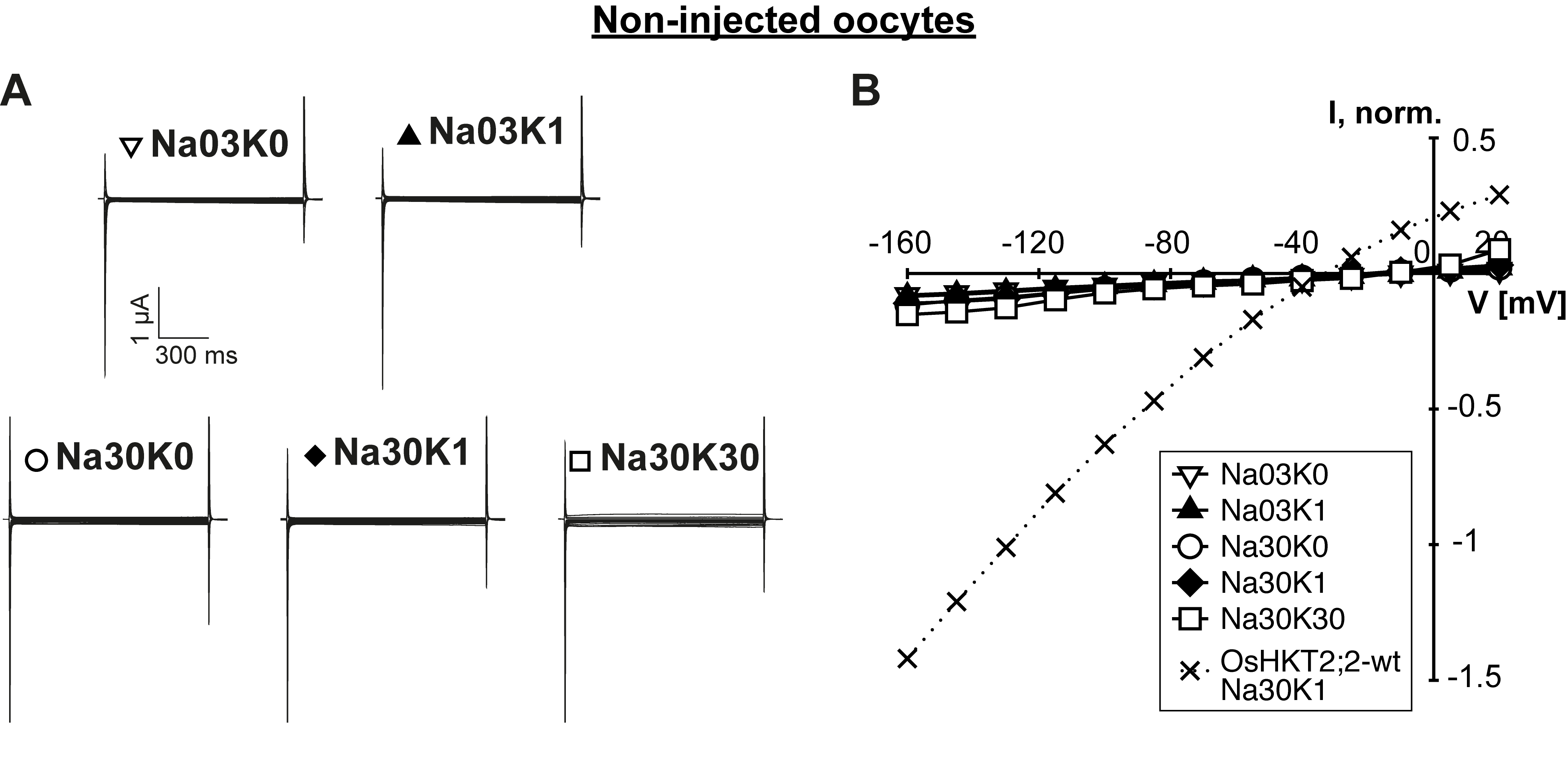

Supplement: Supplementary file 2 — Figure S2. Currents elicited in control cells. (A) Representative currents recorded in Xenopus laevis oocytes at indicated Na+ and K+ concentrations: Na03K0 - 0.3 mM NaCl without KCl, Na03K1 - 0.3 mM NaCl and 1 mM KCl, Na30K0 - 30 mM NaCl without KCl, Na30K1 - 30 mM NaCl and 1 mM KCl, Na30K30 - 30 mM NaCl and 30 mM KCl. Control oocytes underwent the same handling and incubation procedure as injected oocytes and were measured on the same day as cRNA injected oocytes. A pulse at holding potential (zero current level) was followed by 1 s voltage pulses from +20 to -160 mV in -15 mV decrements and continued with a final pulse at holding potential for 1.5 s. (B) Representative current-voltage (IV) curves extracted form current traces shown in (A). For comparison the mean IV curve of OsHKT2;2-wt from Fig. 1g is presented (x). (TIF 574 kb) [file 12870_2019_1909_MOESM2_ESM.tif]

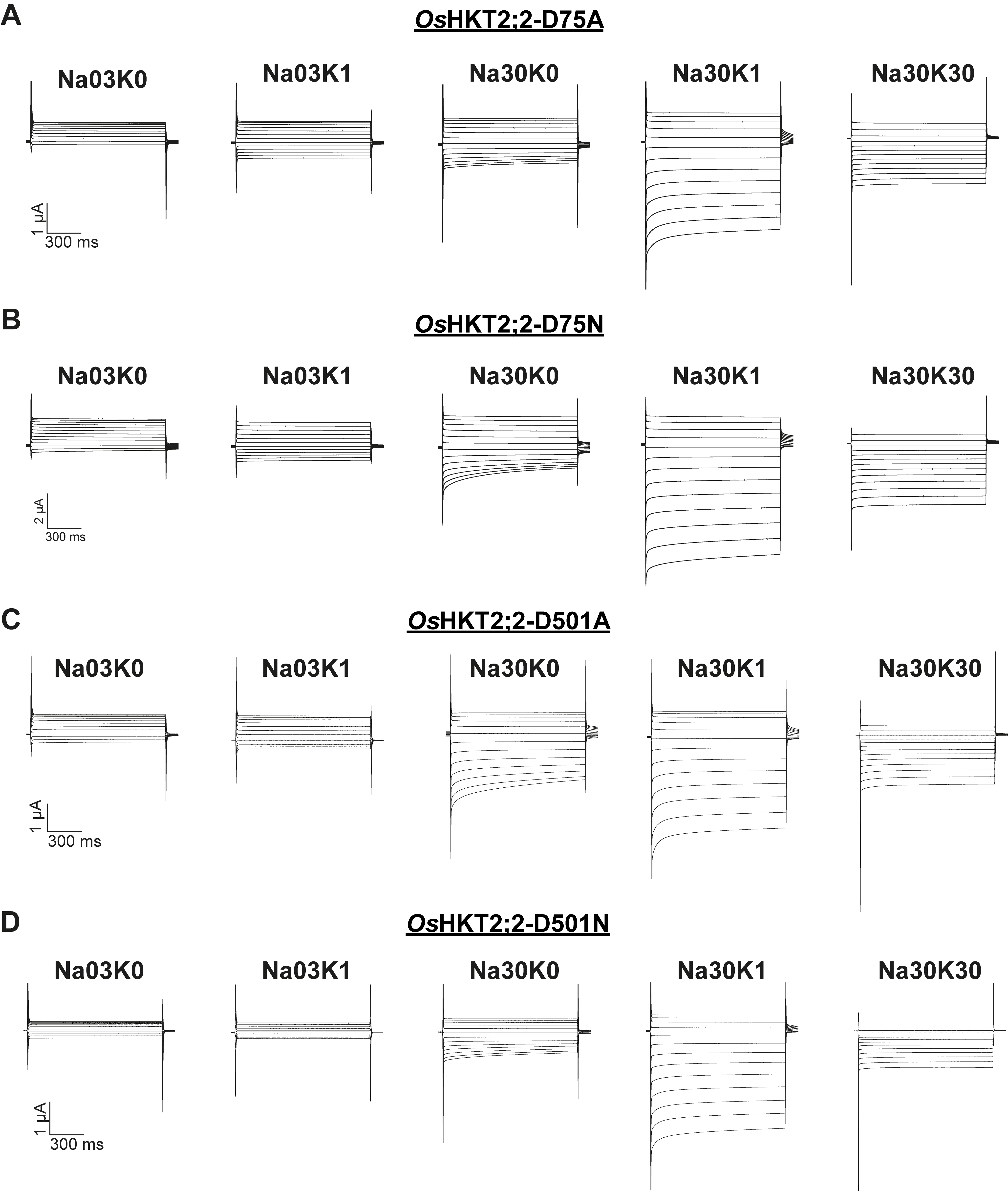

Supplement: Supplementary file 3 — Figure S3. D75A, D75N, D501A and D501N mutants behave comparable to OsHKT2;2-wt. Representative currents recorded in Xenopus laevis oocytes two days after cRNA injection at indicated Na+ and K+ concentrations: Na03K0 - 0.3 mM NaCl without KCl, Na03K1 - 0.3 mM NaCl and 1 mM KCl, Na30K0 - 30 mM NaCl without KCl, Na30K1 - 30 mM NaCl and 1 mM KCl, Na30K30 - 30 mM NaCl and 30 mM KCl. A pulse at holding potential (zero current level) was followed by 1 s voltage pulses from +20 to -160 mV in -15 mV decrements and continued with a final pulse at holding potential for 1.5 s. (TIF 1307 kb) [file 12870_2019_1909_MOESM3_ESM.tif]

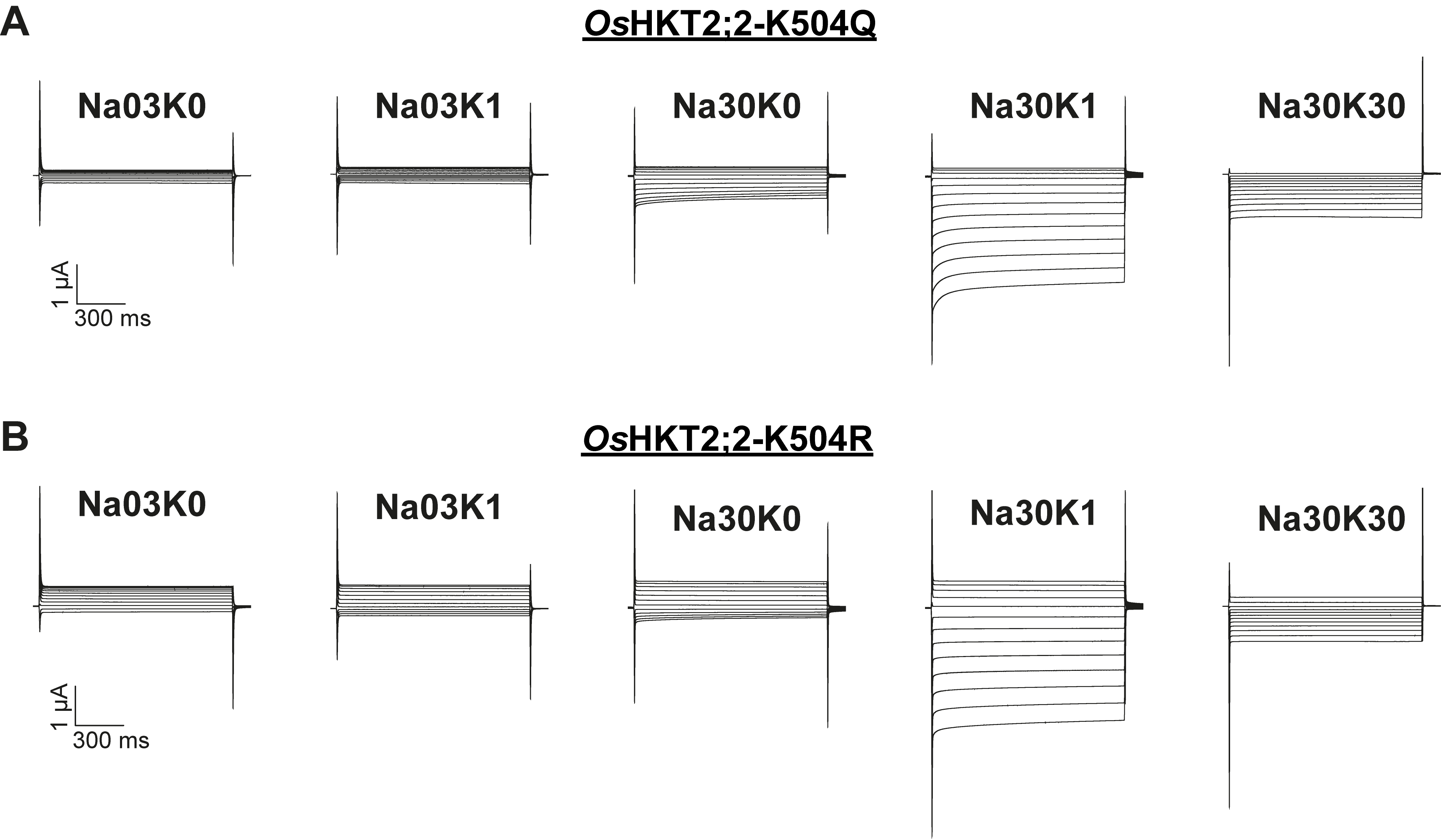

Supplement: Supplementary file 4 — Figure S4. Mutants K504R and K504Q show altered kinetic characteristics in comparison to OsHKT2;2-wt. Representative currents recorded in Xenopus laevis oocytes two days after cRNA injection at indicated Na+ and K+ concentrations: Na03K0 - 0.3 mM NaCl without KCl, Na03K1 - 0.3 mM NaCl and 1 mM KCl, Na30K0 - 30 mM NaCl without KCl, Na30K1 - 30 mM NaCl and 1 mM KCl, Na30K30 - 30 mM NaCl and 30 mM KCl. A pulse at holding potential (zero current level) was followed by 1 s voltage pulses from +20 to -160 mV in -15 mV decrements and continued with a final pulse at holding potential for 1.5 s. (TIF 638 kb) [file 12870_2019_1909_MOESM4_ESM.tif]
